# KIF4A Promotes Glioblastoma Malignant Progression and Transmission of Temozolomide Resistance in the Tumor Microenvironment via the HIF1A/VEGFA Axis

**DOI:** 10.1111/cns.70550

**Published:** 2025-10-13

**Authors:** Xinan Shen, Honglei Cheng, Yihan Xia, Jiarong Zheng, Qiliang Peng, Zhicheng Zhang, Nanheng Yin, Yongshun Liu, Jun Dong, Yuntian Shen

**Affiliations:** ^1^ Department of Radiotherapy & Oncology The Second Affiliated Hospital of Soochow University Suzhou Jiangsu China; ^2^ Institute of Radiotherapy & Oncology Soochow University Suzhou Jiangsu China; ^3^ Suzhou Key Laboratory for Radiation Oncology Suzhou Jiangsu China; ^4^ The First Clinical College, The First Affiliated Hospital Hainan Medical University Haikou Hainan China; ^5^ Department of Obstetrics and Gynecology The Second Affiliated Hospital of Soochow University Suzhou Jiangsu China; ^6^ Department of Neurosurgery The Second Affiliated Hospital of Soochow University Suzhou Jiangsu China

## Abstract

**Background:**

Glioblastoma (GBM) represents the most prevalent primary malignant brain tumor. Temozolomide (TMZ) is the primary chemotherapeutic agent administered following surgical resection. However, the emergence of TMZ resistance in patients undergoing chemotherapy significantly diminishes its efficacy. To date, the majority of preclinical studies have been unsuccessful in effectively overcoming TMZ resistance. Prior research has suggested that the KIF4A gene may serve as a prognostic indicator for patients with GBM. Nevertheless, the precise mechanism by which KIF4A contributes to TMZ resistance remains to be elucidated.

**Methods:**

In order to simulate the in vivo growth environment of glioblastoma, transwell co‐culture and hypoxia induction were employed. Functional experiments were then conducted in order to verify the effects of KIF4A and HIF1A on the proliferation, migration, invasion, and angiogenic ability of GBM cells. Western blot analysis was employed to determine the protein levels of KIF4A, HIF1A, and VEGFA in GBM cells. ELISA was employed to determine the secreted protein levels of VEGFA and MMP9 in GBM cells. A prognostic model was utilized to assess the clinical utility of the pathway. The intercellular resistance to temozolomide was determined through the use of colony formation assays, flow cytometry, and immunofluorescence. The intercellular communication medium and its mechanism were analyzed by electron microscopy, particle size measurement, and WB experiments. Finally, a xenograft tumor model was utilized to investigate the in vivo function of KIF4A.

**Results:**

KIF4A promotes the malignant progression of glioblastoma by regulating the HIF1A/VEGFA axis. GBM cells secrete exosomes to regulate the temozolomide resistance of astrocytes and the expression level of HIF1A/VEGFA protein, thereby making glioblastoma more resistant.

**Conclusion:**

This study provides a new idea for antagonizing temozolomide resistance in glioblastoma. KIF4A promotes malignant progression of glioblastoma and transmission of TMZ resistance in the tumor microenvironment through the HIF1A/VEGFA axis.

## Graphical abstract

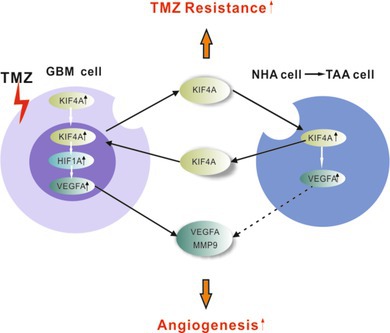

## 1 Introduction

Glioblastoma (GBM) is the most prevalent primary malignant brain tumor [1], with a dismal prognosis and a median survival of approximately 15 months despite aggressive treatment [2]. The standard clinical treatment is surgical resection followed by postoperative chemoradiotherapy with temozolomide [3]. Temozolomide (TMZ) is an oral alkylating agent that readily penetrates the blood–brain barrier [4] with minimal adverse effects and is the first‐line chemotherapeutic agent following surgical resection [5]. However, TMZ resistance frequently occurs in patients in the mid‐ to late‐stage of chemotherapy, which significantly constrains its therapeutic efficacy and results in treatment failure [6]. To date, the majority of preclinical studies have been unsuccessful in effectively overcoming TMZ resistance. Accordingly, further investigation into additional potential mechanisms underlying malignant progression and TMZ resistance in glioblastoma is currently a primary focus of research.

An increasing number of studies have demonstrated that TMZ resistance is not solely a biological phenomenon within GBM cells [7]; rather, it is also influenced by other components of the tumor microenvironment [8]. Astrocytes constitute a substantial component of the glioblastoma tumor microenvironment [9], facilitating intercellular substance transfer and the maintenance of the blood–brain barrier. A complex network of interactions exists between GBM cells and astrocytes, exerting a profound influence on TMZ resistance in glioblastoma [10]. Our previous research has demonstrated that GBM cells are capable of inducing malignant transformation in a wide range of cells within the TME [11]. Nevertheless, the precise mechanism by which TMZ resistance is conveyed within the tumor microenvironment remains unclear.

KIF4A is an essential chromosome‐associated molecular motor located at Xq13.1 in the human genome. It encodes a 140 kDa protein consisting of 1232 amino acids [12]. Prior research has demonstrated that KIF4A functions as an oncogene, facilitating the progression of a range of malignant neoplasms, including bladder [13], esophageal [14], and cholangiocarcinomas [15]. A previous study has found that KIF4A, KIF9, KIF18A, and KIF23 in the KIF family are significantly highly expressed in glioma [16]. By comparing the mRNA and protein expression of the KIF family in glioma and the survival curve of patients, we selected KIF4A with the most obvious difference as the follow‐up research object. In addition, the role of KIF4A in promoting the malignant progression of gliomas through epithelial‐mesenchymal transition (EMT) has been well‐established [17].

The objective of this study was to investigate the role of the KIF4A‐HIF1A‐VEGFA pathway in GBM cells, both intra‐ and intercellularly. This objective was achieved through the utilization of an induced hypoxia and transwell co‐culture system, which has the potential to provide a promising therapeutic strategy for glioblastoma treatment.

## 2 Materials and Methods

### 2.1 Cell Culture

The human GBM cell line GSC23 was obtained from The University of Texas MD Anderson Cancer Centre. The primary SU3 GBM cell line was provided by Prof. Dong Jun of the Second Affiliated Hospital of Soochow University [18]. GSC23 cells were inoculated in DMEM culture flasks containing 10% fetal bovine serum (FBS, Gibco, USA) and 1% penicillin–streptomycin (Gibco Life Technologies, Carlsbad, CA, USA) for a period of 2 weeks in order to induce differentiation. Following this, the cells were incubated in DMEM containing 10% FBS and 1% penicillin–streptomycin in high glucose medium (Gibco Life Technologies, Carlsbad, CA, USA) containing 10% FBS and 1% penicillin–streptomycin. Normal human astrocytes (NHA) (ScienCell, Carlsbad, CA, USA) were cultured in DMEM medium supplemented with 10% FBS and 1% astrocyte growth supplement (ScienCell, USA). The human umbilical vein endothelial cells (HUVEC) were obtained from Cellverse (Shanghai, China) and cultured using a cell‐specific medium (BNCC, Henan, China) designed for use with HUVEC. All cells were maintained in a cell culture incubator at 37°C in a humidified atmosphere containing 5% CO_2_ in accordance with standard laboratory practice. Hypoxia was induced in the cells using an anoxic incubator (Thermo, USA) in a humid atmosphere containing 1% oxygen.

### 2.2 Gene Knockdown and Overexpression

Lentiviral vectors for KIF4A gene overexpression (GV358) and gene knockdown (GV248) were purchased from Genechem (Shanghai, China). The sequences of the shRNAs are presented in Table S1, while the sequences of the siRNAs are shown in Table S2. The lentiviral vectors for KIF4A gene overexpression (GV358) and gene knockdown (GV248) were extracted with RNAiMAX or lipofectamine (Invitrogen, Carlsbad, CA, USA) to transfect GBM cells with siRNAs or lentiviral vectors, in accordance with the manufacturer's instructions.

### 2.3 Bioinformatics Analysis

The single‐cell dataset was obtained from https://cellxgene.cziscience.com/ from Core GBmap under the heading “Harmonized single‐cell landscape, intercellular crosstalk and tumor architecture of glioblastoma.” This was done by first referring to the original text and then annotating the cell populations of the dataset. The localization and quantification of KIF4A, HIF1A, and VEGFA in different single‐cell subpopulations were performed using the R package “scCustomize.” The R packages “GSVA,” “Limma,” “GSEABASE,” and “msigdbr” were used with the Bioconductor parallel computing library “BiocParallel” to enrich for pathway ssGSEA and subsequently generate a heatmap based on KIF4A mRNA expression. A genome enrichment analysis (GSEA) was conducted using the R package “clusterProfiler” to identify subgroup‐specific functionally enriched pathways and tagged genomes. These were extracted from the Molecular Signatures Database (MSigDB, https://software.broadinstitute.org/gsea/msigdb/) to obtain marker (h.all.v7.3) genomes. Enrichment was considered significant if the *p*‐value was less than 0.05.

### 2.4 Cell Counting Kit‐8 (CCK8) Assay

The GBM cell line was inoculated in 96‐well plates with 2000 cells per well and induced in 1% oxygen for 24 h. The original medium was removed, and a 9:1 mixture of 100 μL of medium and CCK‐8 reagent (Vazyme, A311‐01) was added to each well, followed by incubation for 2 h at 37°C. Subsequently, the cells were enumerated using a microplate reader (Vazyme, A311‐01). Subsequently, the absorbance of each sample was quantified at 450 nm using a microplate reader (Biotek, Synergy 2).

### 2.5 Cell Scratch Assay

GBM cells were seeded at 2.5 × 10^5^ cells per well in 6‐well culture plates and incubated for 24 h in an incubator at a low oxygen concentration of 1%. Fully grown cells were scraped evenly with a 200 μL pipette tip, and the medium was replaced with FBS‐free medium. Photographs were taken to record the scratched areas after 0 and 24 h of incubation, respectively. The healing area was analyzed using ImageJ.

### 2.6 Transwell Invasion Test

Matrigel (082706, ABW, China) was dissolved in serum‐free medium at a ratio of 1:8, and then 100 μL was added to each upper chamber. 200 μL of serum‐free medium containing 5 × 10^4^ GBM cells was then added to the upper chamber, and 600 μL of medium containing 10% FBS was added to the lower chamber. The cells were fixed and stained after 24 h of incubation in 1% oxygen.

### 2.7 Tube Formation Assay

Matrigel (Corning, USA) was first thawed at 4°C and then spread into 96‐well plates (50 μL per well) at 37°C for 60 min to form a gel. Different conditioned media were selected to resuspend and inoculate HUVEC (2 × 10^4^/well). After 4 h of incubation, tube formation photographs were taken under a microscope (AMG, USA) and the number of branches was analyzed using ImageJ counting.

### 2.8 ELISA Test

Glioblastoma cell culture medium was collected after different interventions. The secretion of VEGFA and MMP9 was detected using ELISA kits (Abcam, US, AB119566; Enzyme Labeled Biologics, Jiangsu, China, MB‐3558A) according to the manufacturer's instructions. The data were homogenized by counting the number of GBM cells in different groups.

### 2.9 Colony Formation Assay

About 500 GBM cells were inoculated into 6‐well plates and cultured for 24 h. The cells were then treated with DMSO or 200 μM TMZ for 12 days. Cells were fixed in methanol for 15 min and stained with 0.1% crystal violet for 15 min. The cells were observed by microscope and ImageJ software, and the number of clones was counted.

### 2.10 Western Blotting

Cells are lysed with radioimmunoprecipitation assay (RIPA) lysis buffer containing a cocktail of phosphatases and protease inhibitors. A total of 20–30 μg of protein samples was detected by 10% SDS‐PAGE gel and then transferred to a 0.45‐mm polyvinylidene difluoride (PVDF) membrane (Millipore). Membranes were blocked with 5% skimmed milk and incubated with the corresponding primary antibodies overnight at 4°C, followed by incubation with horseradish peroxidase‐conjugated (HRP) secondary antibodies for 1.5 h at room temperature. Finally, membranes were detected using FDbio‐Femto ECL (Fudebio, Hangzhou, China, #FD8000) and chemiluminescence system (Bio‐Rad, Synoptics, Cambridge, UK). The list of antibodies is shown in Table S3.

### 2.11 Cellular Immunofluorescence

Cells affixed to slides were incubated in a hypoxic/normal oxygen incubator for 48 h. After rinsing three times with PBS, the cells were fixed with 4% paraformaldehyde and then left in 0.2% Triton for 10 min to disrupt the cell membrane. 200 μL of blocking solution was added to each well, and the cells were treated for 1 h at room temperature. Cells were treated with anti‐KIF4A antibody (Abclonal, #A10193, 1:200), anti‐HIF1A antibody (ProteinTech, #20960–1‐AP, 1:400), anti‐VEGFA antibody (ProteinTech, #19003–1‐AP, 1:400), and anti‐γH2AX (ProteinTech, #10856–1‐AP, 1:400). Cells were incubated at 4°C overnight. The corresponding primary antibodies were ligated with FITC‐conjugated goat anti‐rabbit IgG (H + L) for 1 h. Observe fluorescence with a laser scanning confocal microscope.

### 2.12 Immunohistochemical Staining

Tumor tissues were collected and fixed in formalin for paraffin embedding. Then the tissue sections (5 μm) were subjected to immunohistochemical staining (anti‐KIF4A dilution of 1:400, anti‐HIF1A dilution of 1:400, anti‐VEGFA dilution of 1:400, anti‐Ki67 dilution of 1:200, and anti‐CD34 antibody dilution of 1:200). IHC images were taken by a hybrid microscope, and the IHC results were evaluated by a semi‐quantitative method. Based on the staining intensity, the overall score is four categories: negative (0), weak (1), moderate (2), and strong (3). The overall score was calculated based on the percentage of positive cells, with the following categories: 1: < 10%; 2: 10%–50%; 3: 51%–80%; and 4: > 80%. The scores from the two categories were aggregated to derive a final score.

### 2.13 Cell Cycle Assay

Cell cycle was detected using a cell cycle kit (Multi Sciences, CCS012). Cells were collected by centrifugation with PBS according to the manufacturer's instructions, and the supernatant was discarded. Add 1 mL of DNA stain and 10 μL of osmotic stabilizer, vortex, and shake to mix, and incubate for 30 min at room temperature away from light. Select the lowest loading rate and analyze using a flow cytometer. Assays were performed in triplicate and statistically analyzed using Flowjo 10.8.1 software.

### 2.14 Exosomes Isolation and Analysis

Exosomes were extracted from the cell culture medium of GSC23 cells through multiple centrifugation procedures with VEX Exosome Isolation Reagent (Vazyme, China). Briefly, the collected culture medium was centrifuged at 2000 rpm for 30 min, then 12,000 rpm for 45 min to remove cell debris and large vesicles. For exosome purification, the supernatant was ultracentrifuged at 100,000*g* for 60 min at 4°C to collect the pellet, then resuspended in 50–100 μL PBS for the subsequent studies.

### 2.15 Tumor Xenograft Assay in Nude Mice

The BALB/C mice (female, 4 weeks, 18 g) used in this study were kept in the SPF standardized animal laboratory of the Animal Centre of the Second Affiliated Hospital of Soochow University. After 1 week of acclimatization, the mice were randomly divided into groups of four mice each. Each mouse was injected subcutaneously with GSC23i or GSC23NC cells (1 × 10^6^ cells/mouse) in the right axilla, and then mice injected with the same cells and carrying tumors of about 50 mm^3^ were treated with TMZ or DMSO (*n* = 4 per group). 20 mg/kg of TMZ was injected intraperitoneally every 2 days in the TMZ group, and an equal amount of DMSO was injected in the DMSO group. 5 days of injection/2 days of withdrawal was used. A 5‐day injection/2‐day withdrawal regimen was used. Tumor volume was measured every 3 days with vernier calipers using the following formula: volume (mm3) = length × width2 × π/6. After treatment, all mice were euthanized, and the tumors were carefully excised, weighed, and photographed. All animal experiments were approved by the Ethics Committee of Soochow University.

### 2.16 Statistical Analysis

All experiments were repeated at least three times, and the results were expressed as mean ± standard deviation. R software (version 4.3.1) and GraphPad Prism 10 were used for data analysis. Comparison of two‐sample means was performed using the independent samples *t*‐test. Comparisons between multiple groups were made using one‐way ANOVA or ANOVA with repeated measures, and further comparisons between the experimental group within a group and the same control group were made using the Dunnett‐*t* test, and two‐by‐two comparisons were made using the Sidak method. Spearman correlation test was used for correlation analysis, and Kaplan–Meier method and Log‐rank test were used for survival analysis. The difference was considered statistically significant at *p* < 0.05.

## 3 Results

### 3.1 KIF4A Regulates the Malignant Phenotype of GBM Cells in a Hypoxic Microenvironment

**FIGURE 1:**
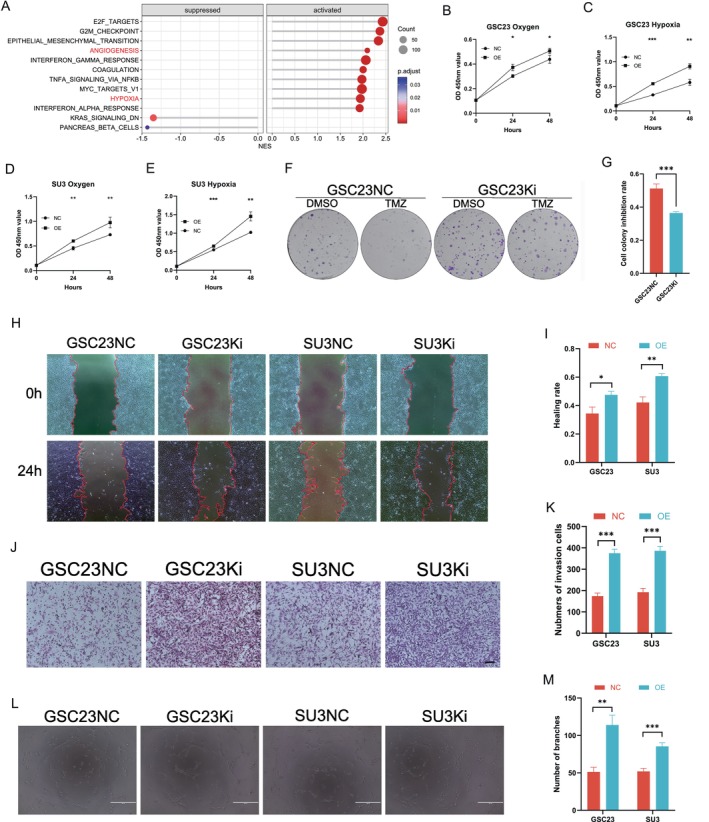
KIF4A regulates the malignant phenotype of GBM cells in a hypoxic microenvironment. (A) GSEA gene set enrichment analysis was used to enrich the pathways related to KIF4A‐associated genes. (B–E) Cell viability of KIF4A overexpression and control groups in hypoxic and normoxic environments by CCK8 assay. OE, overexpression. (F, G) Clonal inhibition of KIF4A overexpression and its control before and after TMZ addition (TMZ concentration 200 μmol). GSC23NC, OE‐vector on GSC23 cell line; GSC23Ki, OE‐KIF4A on GSC23 cell line. (H, I) Wound healing assay detected the migration of GSC23NC cells, GSC23Ki cells, SU3NC cells, and SU3Ki cells under hypoxic conditions. SU3NC, OE‐vector on SU3 cell line; SU3Ki, OE‐KIF4A on SU3 cell line. (J, K) Transwell assay to detect the invasion ability of GSC23NC cells, GSC23Ki cells, SU3NC cells, and SU3Ki cells in hypoxic environment. (L, M) Angiogenesis assay to detect the effect of overexpression of KIF4A on angiogenesis under hypoxic conditions. *p < 0.05, **p < 0.01, ***p < 0.001. *n* = 3 independent experiments. Two‐tailed t test assuming equal variances. Error bars represent the mean ± standard deviation of the mean.

Prior research has indicated that KIF4A is associated with a poor prognosis in patients with glioblastoma [16]. Based on these findings, we hypothesize that KIF4A may promote the malignant progression of glioblastoma through hypoxia and angiogenic pathways. This was determined through gene pathway enrichment analysis (GSEA) (Figure 1A; Figure S1A,B). To facilitate the verification of the impact of KIF4A on the malignant phenotype of GBM cells, we overexpressed KIF4A in SU3 and GSC23 cell lines (Figure S2A). The CCK8 results demonstrated that the cell viability of the KIF4A overexpression group was higher than that of the control group in both normoxia and hypoxia, with a more pronounced proliferation‐promoting effect observed in the absence of hypoxia (Figure 1B–E). Control and KIF4A overexpressing GBM cells were exposed to 200 μmol of TMZ to compare the changes in clone formation rate before and after the addition of KIF4A. The results indicated that the overexpression of KIF4A promoted TMZ resistance in GBM cells (Figure 1F,G). The scratch and transwell invasion assays indicated that the overexpression of KIF4A markedly enhanced the migration and invasion capabilities of GBM cells in a hypoxic environment (Figure 1H–K). The tube‐forming assay results indicated that KIF4A overexpression markedly enhanced angiogenesis within the tumor microenvironment (Figure 1L,M).

### 3.2 Knockdown of KIF4A Inhibits Malignant Progression and Sensitizes GBM Cells to TMZ

To make the argument that KIF4A is involved in regulating the malignant phenotype of GBM cells more convincing, we knocked down KIF4A in SU3, GSC23 cell lines (Figure S2B). CCK8 suggested that knocking down KIF4A significantly inhibited the proliferation of GBM cells in both normoxia and hypoxia (Figure S3A). Cell cloning assays suggested that GBM cells were sensitized to TMZ (Figure S3B,C). Scratch and transwell invasion assays suggested that the knockdown of KIF4A significantly inhibited the migration and invasion ability of GBM cells in a hypoxic environment (Figure S3D–G). The results of the tube‐forming assay suggested that the knockdown of KIF4A inhibited angiogenesis in the tumor microenvironment (Figure S3H–I).

### 3.3 KIF4A Regulates HIF1A/VEGFA Expression in GBM Cells

**FIGURE 2:**
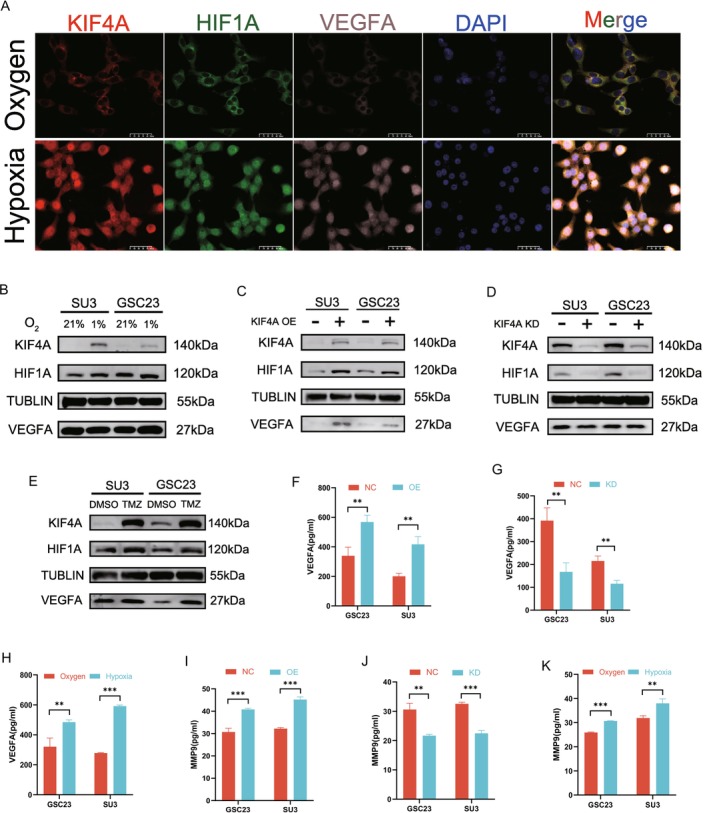
KIF4A regulates HIF1A/VEGFA expression in GBM cells. (A) IF was examined for fluorescence intensity and colocalization of KIF4A, HIF1A, VEGFA in GSC23 GBM cells in hypoxic and normoxic environments. (B–E) WB assay for the protein expression of KIF4A, HIF1A, and VEGFA under the induction of hypoxia, overexpression of KIF4A, knockdown of KIF4A, and addition of TMZ. (F–H) Determination of VEGFA secretory protein concentration in various conditioned media by ELISA assay. (I–K) Determination of MMP9 secretory protein concentration in various conditioned media by ELISA assay. **p < 0.01, ***p < 0.001. *n* = 3 independent experiments. Two‐Tailed *t* test assuming equal variances. Error bars represent the mean ± standard deviation of the mean.

It has been demonstrated that HIF1A and VEGFA are essential targets for regulating the hypoxic microenvironment and neoangiogenesis in GBM cells [19]. On this basis, we propose the scientific hypothesis that KIF4A induces malignant progression and TMZ resistance of glioblastoma in the hypoxic microenvironment by upregulating HIF1A/VEGFA expression in GBM cells. A comparison of the immunofluorescence expression of KIF4A, HIF1A, and VEGFA in GBM cells under normoxic and hypoxic conditions revealed a significant increase in fluorescence intensity for all three proteins in the hypoxic cells, with partial co‐localization (Figure 2A). Furthermore, the main intracellular action sites of the three in hypoxia were observed to shift from extranuclear to intranuclear. The specific mechanism leading to this phenomenon requires further investigation. The aforementioned results indicate that the three proteins exert a synergistic effect on the regulation of the hypoxic microenvironment of GBM cells. The alterations in protein expression of KIF4A, HIF1A, and VEGFA in distinct subgroups of GBM cells following incubation under hypoxic conditions, KIF4A overexpression, KIF4A knockdown, and TMZ treatment were investigated. Western blot analysis demonstrated that the protein expression levels of all three groups were markedly elevated in comparison to the control group following the induction of hypoxia and dosing. Additionally, the protein expression of HIF1A/VEGFA was found to be regulated by KIF4A (Figure 2B–E). Based on this, we further used AlphaFold to predict the possible protein binding sites of KIF4A and HIF1A (Figure S4).

An increasing number of studies have identified tumor neoangiogenesis as an extensive crosstalk between endothelial cells, mural cells, tumor cells, and tumor‐associated immune cells [20]. The expression of VEGFA and MMP9 in the conditioned medium of GBM cells under different culture conditions was detected by ELISA. The findings indicated that the secretion of VEGFA and MMP9 by GBM cells was enhanced under hypoxic conditions and KIF4A overexpression, whereas the knockdown of KIF4A markedly suppressed the secretion of VEGFA and MMP9 by GBM cells (Figure 2F–K).

### 3.4 Knockdown of HIF1A Inhibits Malignant Progression of GBM Cells in Hypoxic Microenvironment and Sensitizes GBM Cells to Chemotherapy

**FIGURE 3:**
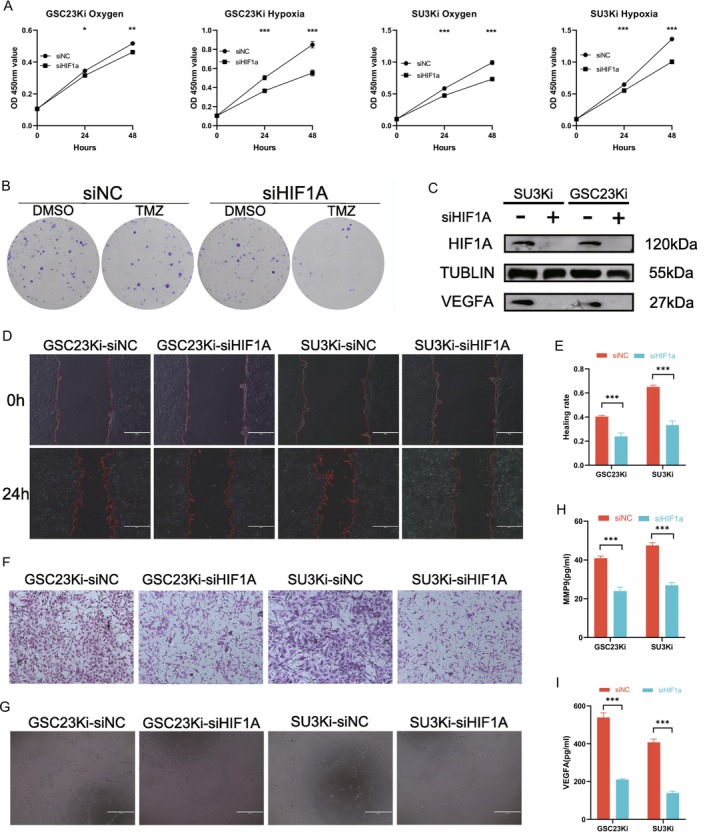
Knockdown of HIF1A inhibits malignant progression of GBM cells in a hypoxic microenvironment and sensitizes GBM cells to chemotherapy. (A) Cell viability of siHIF1A and control groups in hypoxic and normoxic environments by CCK8 assay. (B) Clonal inhibition of siHIF1A and its control before and after TMZ addition (TMZ concentration 200 μmol). (C) Western blotting was performed to detect changes in VEGFA protein expression in HIF1A knockdown GBM cells. (D, E) Wound healing assay detected the migration of various GBM cells under hypoxic conditions. (F) Transwell assay to detect the invasion ability of various GBM cells in a hypoxic environment. (G) Angiogenesis assay to detect the effect of knockdown of HIF1A on angiogenesis under hypoxic conditions. (H‐I) Determination of VEGFA and MMP9 secretory protein concentration in various conditioned media by ELISA assay. *p < 0.05, **p < 0.01, ***p < 0.001. *n* = 3 independent experiments. Two‐tailed t test assuming equal variances. Error bars represent the mean ± standard deviation of the mean.

In order to verify whether KIF4A regulates the malignant phenotype and TMZ resistance of GBM cells through HIF1A, we employed the use of siRNA. To knock down HIF1A in KIF4A overexpression lines, the WB results indicated that the knockdown was successful. The siHIF1A‐1 strand, which demonstrated a superior knockdown effect, was selected for the subsequent experiments (Figure S5A). CCK8 assay revealed that the HIF1A knockdown group significantly inhibited GBM cell proliferation under both normoxia and hypoxia (Figure 3A). Cell cloning assays suggested that the knockdown of HIF1A significantly reversed TMZ resistance caused by the overexpression of KIF4A (Figure 3B; Figure S5B). Scratch and transwell invasion assays suggested that the knockdown of HIF1A significantly inhibited the migration and invasion ability of GBM cells in a hypoxic environment (Figure 3D–F; Figure S5C). The results of the tube‐forming assay suggested that the knockdown of HIF1A significantly inhibited angiogenesis (Figure 3G; Figure S5D). WB assay verified that the knockdown of HIF1A was accompanied by a decrease in the protein expression level of VEGFA (Figure 3C). ELISA assay suggested that the knockdown of HIF1A significantly inhibited the secretion of VEGFA and MMP9 by GBM cells (Figure 3H,I).

### 3.5 Overexpression of HIF1A Promotes the Malignant Progression of GBM Cells in Hypoxic Microenvironment

We used plasmids to overexpress HIF1A in KIF4A stable knockdown GBM cell lines GSC23i and SU3i. Wound healing assay and transwell invasion assay showed that overexpression of HIF1A promoted the migration and invasion of GBM cells under hypoxic conditions (Figure S6A–D). Cell cloning assay showed that overexpression of HIF1A significantly reversed the TMZ resistance induced by KIF4A knockdown (Figure S6E–F). WB results showed that the expression level of VEGFA protein was increased after overexpression of HIF1A (Figure S6G).

### 3.6 Clinical Significance of the KIF4A‐HIF1A‐VEGFA Pathway

**FIGURE 4:**
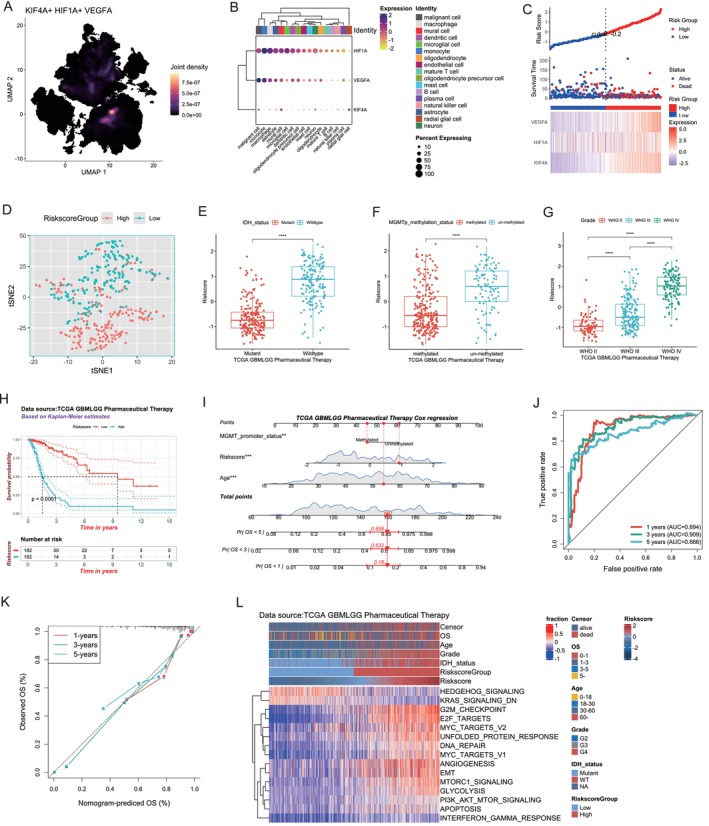
Clinical significance of the KIF4A‐HIF1A‐VEGFA pathway. (A) Dimensional reduction (UMAP) of the gene expression modules (named by colors). (B) Gene expression enrichment of KIF4A, HIF1A, and VEGFA in different cell types. (C) Heat map of KIF4A, HIF1A, and VEGFA gene expression, risk score curve, and scatter plot of survival status in glioma patients treated with chemotherapy. (D) TSNE analysis to determine the clustering performance of the KIF4A‐HIF1A‐VEGFA pathway. (E) Comparison of risk scores for chemotherapy‐treated glioma patients with various IDH status. (F) Comparison of risk scores for chemotherapy‐treated glioma patients with various MGMT‐methylation status. (G) Comparison of risk scores for chemotherapy‐treated glioma patients with various WHO grades. (H) K‐M analysis of glioma patients treated with chemotherapy in the high‐risk and low‐risk groups. (I) A Nomogram was constructed to predict 1‐, 3‐, and 5‐year survival of glioma patients based on three independent prognostic factors (risk score, age and MGMT‐promoter status). (J) ROC curve analysis of the nomogram. (K) Calibration plots to verify the accuracy of the nomogram. (L) GSVA analysis of activation pathways in the high‐risk and low‐risk groups. TSNE, T‐Distributed Stochastic Neighbor Embedding; ROC, Receiver operating characteristic curve; AUC, Area under the curve; **p < 0.01, ***p < 0.001, ****p < 0.0001. *n* = 3 independent experiments. Two‐Tailed t test assuming equal variances. Error bars represent the mean ± standard deviation of the mean.

Subsequent to the identification of the KIF4A‐HIF1A‐VEGFA pathway, further bioinformatics analyses were conducted to investigate the prognostic significance of these three genes. The ‘Core GBmap’ single‐cell dataset, derived from the cellX‐gene database, was employed to co‐localize KIF4A, HIF1A, and VEGFA. This finding validated the hypothesis that all three genes interact synergistically with KIF4A‐HIF1A‐VEGFA pathways in GBM malignant cells, as they were all expressed in malignant cell families (Figure 4A,B). The Cox proportional risk model was constructed using the R package (ggrisk) and the transcript levels of the three genes (KIF4A, HIF1A, and VEGFA) in the dataset, as well as clinical prognostic data, in order to calculate the risk scores and risk factor linkage maps (Figure 4C). As the risk score increased, there was a corresponding increase in the transcript levels of KIF4A, HIF1A, and VEGFA. TSNE analysis classified the samples into two distinct categories (Figure 4D). The IDH wild‐type group was found to exhibit worse prognostic features [21]. A recent study in patients with GBM multiforme have demonstrated that hypermethylation of the MGMT promoter is associated with improved overall survival (OS) and a more significant response to TMZ therapy [22]. The statistical results of the glioma patient samples within the risk model indicated that patients with higher risk scores were more likely to exhibit IDH wild‐type and MGMT promoter demethylation (Figure 4E,F), which is consistent with the findings of our previous in vitro experiments. There was a positive correlation between the grade of glioma and the risk score, with WHO IV samples exhibiting the highest risk score (Figure 4G). The dataset was divided into high‐ and low‐risk groups based on the median risk score, and a Kaplan–Meier survival analysis demonstrated that patients in the high‐risk group exhibited significantly reduced OS compared to those in the low‐risk group (Figure 4H). To facilitate visualization of this prognostic model, a column line plot was constructed, combining the risk score, age, and MGMT promoter expression of the dataset to predict the risk of death at 1, 3, and 5 years for each patient (Figure 4I). For example, for a 54‐year‐old standard treated glioblastoma patient with MGMT methylation and a risk score of 1, the prognostic model suggested that the patient's risk of death at 1, 3, and 5 years was 0.16, 0.532, and 0.858, respectively. ROC curve analysis demonstrated that the AUC value corresponding to 1‐, 3‐, and 5‐year survival was approximately 0.9, indicating the sensitivity and specificity of the column line plot in predicting patient survival rates (Figure 4J). The calibration plots of each fitted line exhibited a near‐overlap with the ideal curve, thereby reflecting the accuracy of the column line plot prediction (Figure 4K). By analyzing the Chinese Glioma Gene Expression Atlas (CGGA) dataset as an external cohort, the prognostic value and wide applicability of this pathway were further demonstrated (Figure S7). Subsequently, we conducted a gene set variation analysis (GSVA) on the high‐ and low‐risk subgroups of the samples to elucidate the pathways that were enriched in the model. The pathway heatmap demonstrated that DNA damage repair, E2F, and EMT‐related molecular pathways were enriched in the high‐risk group (Figure 4L).

### 3.7 The KIF4A‐HIF1A‐VEGFA Pathway Is Implicated in the Transfer of TMZ Resistance Between Cells Within the Tumor Microenvironment

**FIGURE 5:**
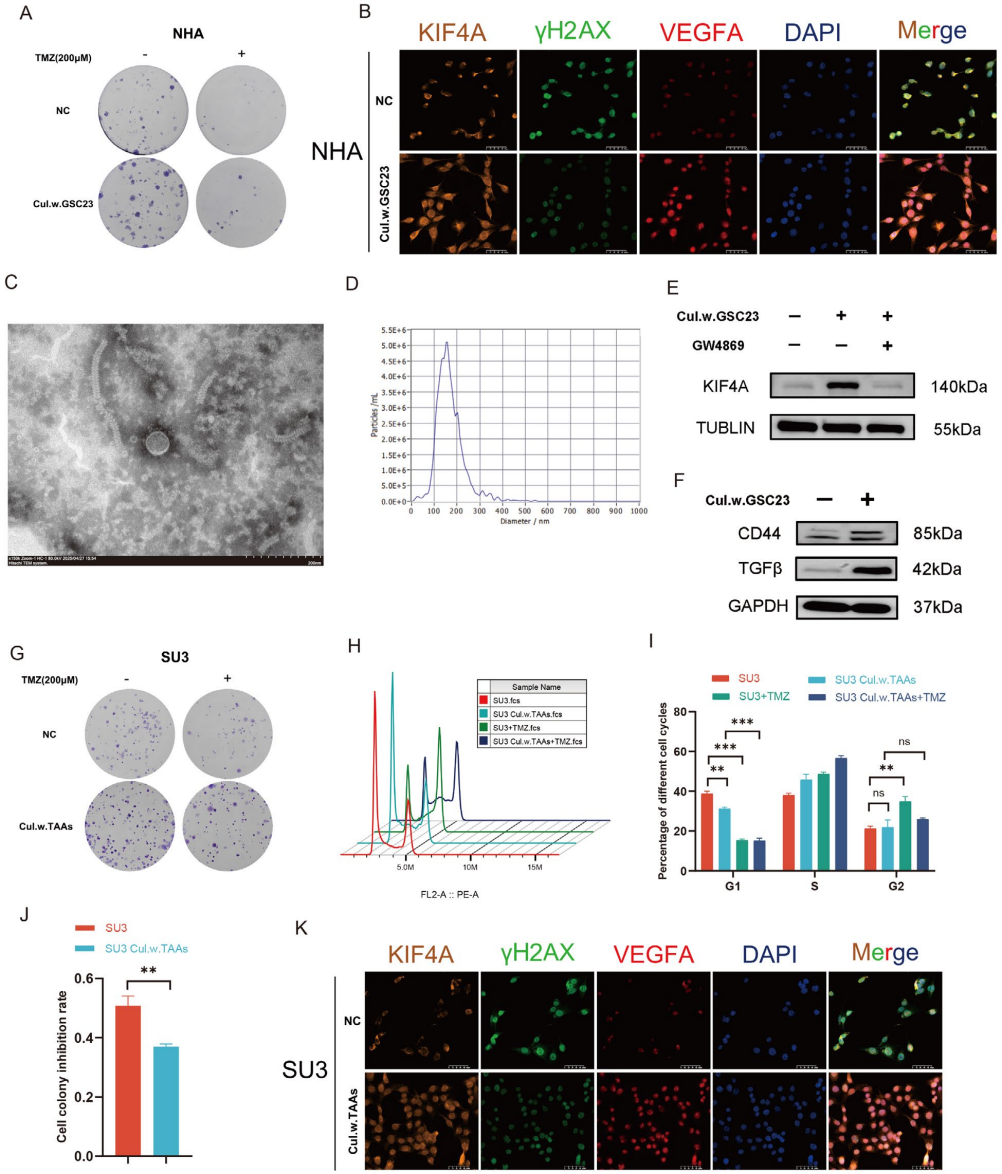
The culture and control groups before and after TMZ treatment aspathway is implicated in the transfer of TMZ resistance between cells within the tumor microenvironment. (A) Clonogenic inhibition of GSC23 co‐culture and control groups before and after TMZ treatment as determined by cell cloning assay. (B) Comparison of KIF4A, γH2AX, and VEGFA expression in GSC23 co‐culture and control groups by immunofluorescence assay. (C) Transmission electron microscopy of exosomes derived from GBM cells. (D) Size distribution analysis of exosomes derived from GBM cells. (E) Western blotting was used to detect the protein expression of KIF4A in the NHA group, GSC23 co‐culture group, and GSC23 co‐culture with GW4869. (F) Western blotting was used to detect the protein expression of CD44 and TGFβ in the NHA group and GSC23 co‐culture group. (G, J) Clonogenic inhibition of TAA co‐culture and control groups before and after TMZ treatment as determined by cell cloning assay. (H, I) Flow cytometry detection of cell cycle changes in TAA co‐culture and control groups before and after the addition of TMZ. (K) Comparison of KIF4A, γH2AX, and VEGFA expression in TAA co‐culture and control groups by immunofluorescence assay. **p < 0.01, ***p < 0.001. *n* = 3 independent experiments. Two‐tailed t test assuming equal variances. Error bars represent the mean ± standard deviation of the mean.

The tumor microenvironment (TME) plays a key role in several aspects of glioma progression, especially in TMZ resistance [23]. In gliomas, astrocytes and microglia represent significant elements of the TME [24]. It is frequently observed that glioma and non‐tumor cells exchange information in contact‐independent ways, such as through the secretion of exosomes and the release of extracellular vesicles [25]. To eliminate the possibility of contact‐dependent cellular communication, a noncontact co‐culture of NHA with GSC23 cells was employed in this experiment. The GSC23 cells were inoculated in the upper chamber, while the NHA cells were inoculated in the lower chamber. The two were then co‐cultured for a period of 5 days before TMZ was added to assess any changes in TMZ resistance in the NHA cells. The results of the cell cloning experiment indicated that the TMZ resistance of NHA cells following co‐culture with GSC23 cells was markedly elevated in comparison to the control group (Figure 5A; Figure S8A). The results of the cell cycle analysis demonstrated a notable reduction in the proportion of cells in the G1 phase and an increase in those in the G2 phase in the co‐culture group relative to the control group. This suggests that the proliferation and viability of the cells were enhanced in the former, and that the G2/M phase block was diminished in the co‐culture group in comparison to the control group, both prior to and following the introduction of the drug, indicating that the NHA cells exhibited heightened resistance to the drug in the co‐culture group relative to the previous scenario (Figure S8B,C). The immunofluorescence results demonstrated that the fluorescence expression of KIF4A and VEGFA was elevated and that of γ‐H2AX was significantly reduced in the co‐culture group relative to the control group after the addition of an equivalent dose of TMZ (Figure 5B). To further clarify the mechanism of intercellular interaction, exosomes were isolated from the conditioned medium of GSC23, and electron microscopy and particle size measurement were performed. A typical lipid bilayer was observed in the purified exosomes, and the size of the exosomes was consistent with the standard range confirmed by TEM and NTA (Figure 5C,D). We further detected the expression of KIF4A protein in the NHA group, GSC23 co‐culture group, and GSC23 co‐culture +GW4869 group. The results showed that the expression level of KIF4A protein in the GSC23 co‐culture group was significantly higher than that in the NHA group. However, the expression level of KIF4A protein in the GSC23 co‐culture +GW4869 group was significantly lower than that in the GSC23 co‐culture group (Figure 5E). We detected the protein expression of tumor‐related markers CD44 and TGFβ in NHA cells and the GSC23 co‐culture group by WB experiment, and the results showed that the tumor markers in the GSC23 co‐culture group were significantly higher than those in the control group (Figure 5F). The above results suggest that KIF4A is transferred to NHA cells via exosomes secreted by GBM cells, which renders NHA cells resistant to TMZ and transforms them into tumor‐associated astrocytes (TAAs). On this basis, we proceeded to investigate whether TAA cells could transfer TMZ resistance to GBM cells. The results of the cell cloning experiment indicated that TMZ resistance was markedly elevated in the co‐culture group in comparison to the control group (Figure 5G,J). The cell cycle results demonstrated a reduction in the number of G1 phase cells in the co‐culture group in comparison to the control group. Furthermore, the G2/M phase block was observed to be significantly lower in the co‐culture group, both before and after the addition of the drug, in comparison to the control group. This suggests that SU3 cells exhibited heightened resistance to the drug following co‐culture, in comparison to prior to co‐culture (Figure 5H,I). Immunofluorescence analysis demonstrated that the expression of KIF4A and VEGFA was elevated and that of γ‐H2AX was diminished in the co‐culture group relative to the control group following the administration of an equivalent dose of TMZ (Figure 5K). The aforementioned results indicated that TAAs may facilitate the delivery of KIF4A to GBM cells via noncontact mechanisms, thereby contributing to the enhancement of TMZ resistance in GBM cells.

### 3.8 Knockdown of KIF4A Increases the Sensitivity of Glioblastoma to TMZ in Nude Mice

**FIGURE 6:**
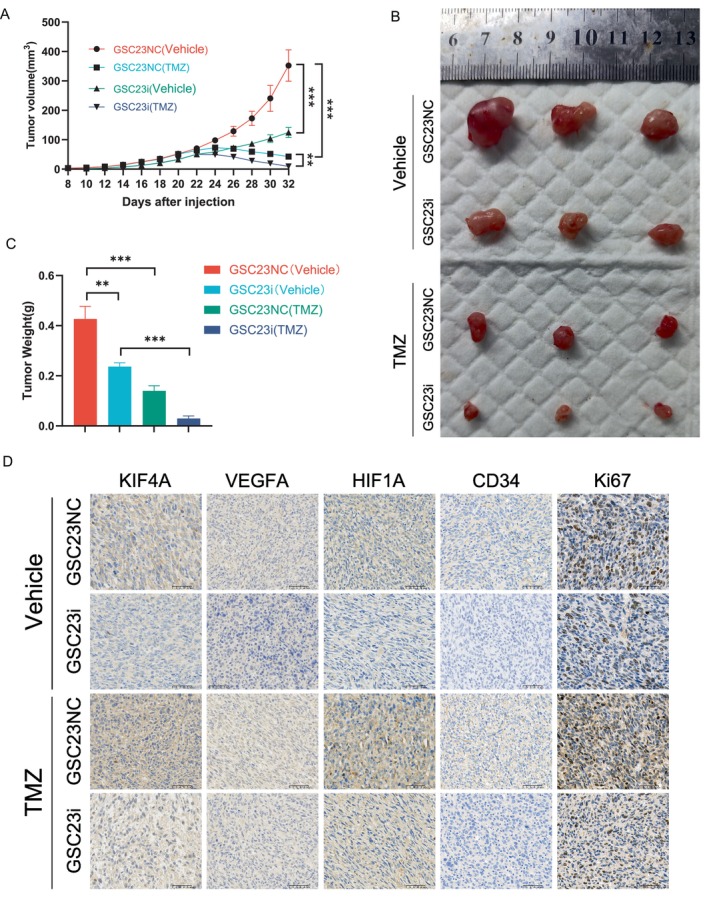
Knockdown of KIF4A increases the sensitivity of glioblastoma to TMZ in nude mice. (A) Growth curves of tumor volume in mice. (B, C) Size and weight of tumors isolated from nude mice. (D) Representative images of IHC staining for KIF4A, HIF1A, VEGFA, CD34, and Ki67 in 4 groups of mice. GSC23i, KD‐KIF4A on GSC23 cell line; GSC23NC, KD‐vector on GSC23 cell line. **p < 0.01, ***p < 0.001. *n* = 3 independent experiments. Two‐tailed *t* test assuming equal variances. Error bars represent the mean ± standard deviation of the mean.

In order to investigate the role of KIF4A in regulating TMZ sensitivity in vivo, a nude mouse xenograft model was constructed. Subcutaneous implantation of GSC23i cells or GSC23NC cells was performed in nude mice, with subsequent monitoring of the transplanted tumor growth. Once the tumor volume had reached 50 cubic mm, the mice were administered TMZ/DMSO via intraperitoneal injection. Tumor volume was monitored in real time, and it was observed that the rate of tumor growth in the TMZ treatment group was significantly reduced or even subsided compared to the previous measurement, indicating that TMZ has an anti‐tumor effect on mice. The tumor volume of mice in the KIF4A knockdown group was observed to be smaller than that of the control group when treated with TMZ/DMSO (Figure 6A). The final isolation also indicated that the knockdown of KIF4A markedly impeded the tumor‐forming capacity of GSC23 GBM cells in mice (Figure 6B,C). A comparison of the immunohistochemical staining results for KIF4A, HIF1A, VEGFA, CD34, and Ki67 in the different groups revealed a significant reduction in protein expression in the knockdown group relative to the control group. Conversely, protein expression in the additive group was increased relative to the control group (Figure 6D). These findings suggest that the knockdown of KIF4A may inhibit the in vivo tumorigenicity and malignancy of GBM cells, while enhancing the sensitivity of tumors to TMZ.

## 4 Discussion

TMZ is currently the primary pharmacological agent utilized in the clinical management of glioblastoma [26], which has the potential to prolong survival in the majority of glioma patients [27]. However, due to the significant drug resistance observed in many glioblastoma patients, the prognosis for survival remains unsatisfactory, according to clinical workers [28]. Consequently, identifying the mechanism that can sensitize or even reverse TMZ resistance has become a pressing issue that requires urgent resolution.

Prior research has demonstrated that the malignant progression of glioma and TMZ resistance is not an independent biological behavior of GBM cells [29]. Instead, non‐tumor cells and pro‐angiogenic cells within the tumor microenvironment play a significant role [30]. Astrocytes have been identified as a key area of focus in our research due to their substantial cellular population and intricate biological functions [31]. Although previous studies have indicated that GBM cells may contribute to the malignant progression of astrocytes [32], the intrinsic mechanism of crosstalk between these two cell types that promotes increased TMZ resistance remains to be further elucidated.

A substantial body of literature demonstrates that kinesin superfamily genes (KIF) facilitate the malignant progression of gliomas [16]. By analyzing the paraneoplastic mRNA and protein expression as well as survival curves of different genes within the family, we found that KIF4A exhibited the most significant differential expression. Prior research has indicated that KIF4A plays a pivotal role in cell cycle regulation [33]. The present study demonstrated that KIF4A facilitates the transition from G1 to G2 phase in GBM cells and effectively antagonizes the G2/M phase block induced by the action of TMZ drugs. The HIF1A/VEGFA axis plays a pivotal role in the malignant progression of glioblastomas [34]. In contrast to peritumoral tissues, where HIF1A/VEGFA is markedly overexpressed within the tumor, D'Alessio's study demonstrated that HIF1A/VEGFA axis expression is positively correlated with glioma histological typing [35]. Conversely, Korkolopoulou's study indicated that, in contrast to peritumoral tissues, HIF1A/VEGFA is significantly overexpressed within the tumor [36]. It has been postulated that TMZ may activate the stress mechanism of HIF1A expression, which in turn upregulates VEGFA expression, thereby resulting in a poor prognosis for glioma patients [37]. Bevacizumab plays a significant role in the treatment of recurrent glioblastoma due to its anti‐VEGF effect [38]. Nevertheless, the current anti‐VEGF monotherapy, including bevacizumab, has not demonstrated efficacy in improving OS rates [39]. In this study, we found that knockdown of KIF4A significantly downregulated the HIF1A/VEGFA pathway in glioma cells and the tumor microenvironment. Small molecule drugs targeting KIF4A are expected to provide a new treatment strategy for patients with temozolomide resistance and bevacizumab sensitivity failure in clinical treatment.

The present study verified that KIF4A promotes malignant progression of GBM cells through the HIF1A/VEGFA axis in a hypoxic microenvironment. KIF4A facilitates glioblastoma neoangiogenesis by promoting the secretion of VEGFA and MMP9. Additionally, KIF4A can mediate the crosstalk between GBM cells and astrocytes through exosomes to stimulate the up‐regulation of HIF1A/VEGFA, which enhances chemoresistance in glioblastoma. We propose and validate, for the first time, that KIF4A promotes malignant progression of glioblastoma and cell‐to‐cell transmission of TMZ resistance through the HIF1A/VEGFA axis. This provides a new therapeutic idea for patients with subsequently relapsed and TMZ‐resistant glioblastoma.

## 5 Conclusions

In conclusion, KIF4A has been demonstrated to facilitate malignant progression and TMZ resistance in GBM cells via the HIF1A/VEGFA axis. KIF4A plays a pivotal role in mediating crosstalk between GBM cells and astrocytes, thereby regulating intercellular transmission of TMZ resistance. This provides a theoretical basis for subsequent chemotherapy sensitization of glioblastoma.

## Author Contributions

X.S. was responsible for conception, method validation, and writing‐original; H.C., Y.X., Q.P. were responsible for formal analysis; X.S., H.C., Y.X., J.Z., Z.Z., N.Y., and Y.L. were responsible for resources and data compilation; Y.S. and J.D. were responsible for study design and writing‐review and editing. All authors read and approved the final manuscript.

## Consent

Consents to publish this paper were available from all authors.

## Conflicts of Interest

The authors declare no conflicts of interest.

## Supporting information


**Data S1:** Supporting Information.

## Data Availability

The data that support the findings of this study are available from the corresponding author upon reasonable request.
