# Supplementary material for: KIF4A Promotes Glioblastoma Malignant Progression and Transmission of Temozolomide Resistance in the Tumor Microenvironment via the HIF1A/VEGFA Axis
Source: CNS Neurosci Ther. 2025 Oct 13;31(10):e70550. doi: 10.1111/cns.70550 (PMC12518161; doi:10.1111/cns.70550)
Supplement: Supplementary file 1 — Data S1: Supporting Information. [file CNS-31-e70550-s001.zip › Supplementary information.doc]

**KIF4A promotes malignant progression of glioblastoma and transmission of TMZ resistance in the tumour microenvironment via the HIF1A/VEGFA axis**

A	B

**Supplementary Figure 1. KIF4A significantly enriched in hypoxia and angiogenesis pathways.**

(A-B) GSEA analysis of protein processing in hypoxia and angiogenesis pathways.

A	B

**Supplementary Figure 2. Validation of knockdown and overexpression in cell lines.**

(A-B) Western blotting to validate the knockdown and overexpression of KIF4A.

**Supplementary Figure 3. Knockdown of KIF4A inhibits malignant progression and sensitizes GBM cells to TMZ**

(A) Cell viability of KIF4A knockdown and control groups in hypoxic and normoxic environments by CCK8 assay. KD, Knockdown. (B-C) Clonal inhibition of KIF4A knockdown and its control before and after TMZ addition(TMZ concentration 200 μmol). (D-E) Wound healing assay detected the migration of GSC23NC cells, GSC23i cells, SU3NC cells, and SU3i cells under hypoxic conditions. GSC23NC, KD-vector on GSC23 cell line; GSC23i, KD-KIF4A on GSC23 cell line; SU3NC, KD-vector on SU3 cell line; SU3i, KD-KIF4A on SU3 cell line. (F-G) Transwell assay to detect the invasion ability of GSC23NC cells, GSC23i cells, SU3NC cells, and SU3i cells in hypoxic environment. (H-I) Angiogenesis assay to detect the effect of knockdown of KIF4A on angiogenesis under hypoxic conditions. *P < 0.05, **P < 0.01, ***P < 0.001. n = 3 independent experiments. Two-Tailed t test assuming equal variances. Error bars represent the mean ± standard deviation of the mean.

**Supplementary Figure 4. AlphaFold was used to predict the possible binding sites of the KIF4A and HIF1A proteins.**

A	B

C	D

**Supplementary Figure 5. Knockdown of HIF1A inhibits malignant progression of GBM cells in a hypoxic microenvironment and sensitizes GBM cells to chemotherapy.**

(A) Validation of HIF1A knockdown efficiency by Western blotting. (B) Clonal inhibition of HIF1A knockdown and its control before and after TMZ addition (TMZ concentration 200 μmol). (C) Transwell assay to detect the invasion ability of HIF1A knockdown and its control in hypoxic environment. (D) Angiogenesis assay to detect the effect of knock down of HIF1A on angiogenesis under hypoxic conditions. ***P < 0.001. n = 3 independent experiments. Two-Tailed t test assuming equal variances. Error bars represent the mean ± standard deviation of the mean.

**Supplementary Figure 6. Overexpression of HIF1A promotes the malignant progression of GBM cells in hypoxic microenvironment**

(A-B) Wound healing assay detected the migration of GSC23i-OENC cells, GSC23i-OEHIF1A cells, SU3i-OENC cells, and SU3i-OEHIF1A cells under hypoxic conditions. (C-D) Transwell assay to detect the invasion ability of GSC23i-OENC cells, GSC23i-OEHIF1A cells, SU3i-OENC cells, and SU3i-OEHIF1A cells in hypoxic environment. (E-F) Clonal inhibition of KIF4A overexpression and its control before and after exposure to TMZ (TMZ concentration 200 μmol). (G) Western blotting to validate the overexpression of HIF1A. *P < 0.05, **P < 0.01, ***P < 0.001. n = 3 independent experiments. Two-Tailed t test assuming equal variances. Error bars represent the mean ± standard deviation of the mean.

**Supplementary Figure 7. Heat map of KIF4A, HIF1A and VEGFA gene expression, risk score curve and scatter plot of survival status in glioma patients treated with chemotherapy.**

A	B	C

**Supplementary Figure 8. The KIF4A-HIF1A-VEGFA pathway is implicated in the transfer of TMZ resistance between cells within the tumor microenvironment**

(A-B) Flow cytometry detection of cell cycle changes in GSC23 co-culture and control groups before and after exposure to TMZ. (C) Clonogenic inhibition of GSC23 co-culture and control groups before and after TMZ treatment as determined by cell cloning assay. *P < 0.05, **P < 0.01, ***P < 0.001. n = 3 independent experiments. Two-Tailed t test assuming equal variances. Error bars represent the mean ± standard deviation of the mean.

**Supplementary Table 1. KIF4A sh**RNA targets Sequences

| **shRNA** | **Oligo** |
| --- | --- |
| KIF4A KD#1 | TACTGCAGAGCAAGAGAATGA |
| KIF4A KD#2 | GAGCTGGTTGAGTTGAATAAA |
| KIF4A KD#3 | CCAGATACCAGCAGGTCTT |

**Supplementary Table 2. HIF1A siRNA tar**gets Sequences

| **siRNA** | **Oligo** |
| --- | --- |
| HIF1A si-1 | GACGAUCAUGCAGCUACUACA  UAGUAGCUGCAUGAUCGUCUG |
| HIF1A si-2 | CGAUGGAAGCACUAGACAAA  UUGUCUAGUGCUUCCAUCGGA |
| HIF1A si-3 | GAUCAGUUGUCACCAUUAGA  UAAUGGUGACAACUGAUCGAA |

**Supplementary Table 3. List of antibodies**

| **Antibodies** | **Source** | **Identifer** |
| --- | --- | --- |
| KIF4A | Abclonal Technology | A10193 |
| HRP-conjugated Goat anti-Rabbit IgG (H+L) | Abclonal Technology | AS014 |
| HRP-conjugated Goat anti-Mouse IgG (H+L) | Abclonal Technology | AS003 |
| TUBLIN | ProteinTech Group | 10094-1-AP |
| HIF1A | ProteinTech Group | 20960-1-AP |
| VEGFA | ProteinTech Group | 19003-1-AP |
| CD34 | ProteinTech Group | 14486-1-AP |
| γH2AX | ProteinTech Group | 10856-1-AP |
| Ki67 | Aifang Biological | AF300540 |
| CD44 | ProteinTech Group | 15675-1-AP |
| TGFβ | ProteinTech Group | HZ-1011 |
