# Supplementary material for: KIF4A Promotes Glioblastoma Malignant Progression and Transmission of Temozolomide Resistance in the Tumor Microenvironment via the HIF1A/VEGFA Axis
Source: CNS Neurosci Ther. 2025 Oct 13;31(10):e70550. doi: 10.1111/cns.70550 (PMC12518161; doi:10.1111/cns.70550)
Supplement: Supplementary file 1 — Data S1: Supporting Information. [file CNS-31-e70550-s001.zip › Uncropped western blots.doc]

**Uncropped western blots**

**A**

**B**

**C**

**D**

**E**

**F**

**G**

**H**

**I**

**(A)** Uncropped western blots for main Figure 2B. **(B)** Uncropped western blots for main Figure 2C,Supplementary Figure 2A. **(C)** Uncropped western blots for main Figure 2D,Supplementary Figure 2B. **(D)** Uncropped western blots for main Figure 2E. **(E)** Uncropped western blots for Supplementary Figure 4A. **(F)** Uncropped western blots for main Figure 3C. **(G)** Uncropped western blots for main Figure 5F. **(H)** Uncropped western blots for main Figure 5E. **(I)** Uncropped western blots for Supplementary Figure 6G.
